# Using the validated Reflective Functioning Questionnaire to investigate mentalizing in individuals presenting with eating disorders with and without self-harm

**DOI:** 10.7717/peerj.5756

**Published:** 2018-10-29

**Authors:** Angie Cucchi, James A. Hampton, Alesia Moulton-Perkins

**Affiliations:** 1 Department of Psychology, City, University of London, London, England, United Kingdom; 2 Department of Psychology, University of Surrey, Guildford, England, United Kingdom

**Keywords:** Mentalizing, Self-harm, Eating-disorders, Reflective-function, Hyper/hypo-mentalizing, Comorbidity

## Abstract

**Background:**

The present study builds on previous research which explored the relationship between mentalizing and eating disorders (ED) in a subgroup of patients with comorbid self-harm (SH). Whereas previous literature had linked this comorbidity to impulse-control difficulties, more recent advances have suggested that a lack of a mentalizing stance might be responsible for a more treatment-resistant and severe symptomatology in this subgroup of clients.

**Methods:**

A cross-sectional, quasi-experimental, questionnaire-based, between-groups design was employed and a measure of mentalizing was compared in individuals presenting with ED only, individuals presenting with ED and concurrent SH and a control group.

**Results:**

Individuals with ED with concurrent SH reported significantly more mentalizing ability impairment than individuals without concurrent SH. In addition, both groups differed significantly from the control group. Opposite scoring patterns were identified in hypo- and hypermentalizing with the comorbid group reporting the lowest scores in hypermentalizing and the highest scores in hypomentalizing.

**Conclusions:**

The current findings confirm that individuals with concurrent ED and SH report more severe impairments in mentalizing ability. Such impairments entail difficulties in symbolic capacity and abstract thinking and a concretisation of inner life, exemplified by a rigid, often inflexible focus on the physical world. The clinical implications that a lack of a mentalizing stance can have on individuals’ ability to engage with the therapeutic process and to initiate change are reflected upon.

## Introduction

Mentalization, also known as “reflective function” is defined as ‘*the mental process by which an individual implicitly and explicitly interprets the actions of himself and others as meaningful on the basis of intentional mental states* (Bateman & Fonagy, 2004). Its measurement has until recently been accomplished using the Reflective Function Rating Scale (Fonagy et al., 1998), an interview-based method requiring extensive training and only producing a unidimensional scale for what many have argued may be a multi-dimensional concept (Gunderson & Choi-Kain, 2008). Desiring a more practical and less labour-intensive tool, Fonagy and colleagues developed the self-report Reflective Function Questionnaire, RFQ (Fonagy et al., 2016). Cucchi (2016) used a pilot pre-validated version of this questionnaire to show impairments in mentalizing ability in individuals presenting with ED compared to a control group. However, in spite of a marginally significant trend, no firm conclusions could be reached about whether ED individuals who present with concurrent SH have significantly lower mentalizing abilities than those without concurrent SH. While the final RFQ was validated on a similar sample to that used by Cucchi, the pre-validated version featured greater length (54 items versus 8), a distinct scoring system (mixed polar and median-scored items versus a simplified polar scoring system) and a lack of subscales, features which may have constrained its ability to detect subtle mentalizing differences. With the publication of the final validated version of the RFQ (Fonagy et al., 2016), an opportunity has been provided to re-analyse and verify Cucchi’s (2016) preliminary findings. In addition, the recent version of the RFQ, with the two newly developed subscales assessing Certainty (RFQc) and Uncertainty (RFQu) about mental states, which aim to measure hypermentalizing and hypomentalizing respectively (Fonagy et al., 2016), provides an opportunity to further explore whether individuals who present with ED and SH differ in terms of hypo- or hyper-mentalizing. Hypomentalizing has been defined as extreme difficulty in developing complex models of the minds of others and/or the self and has been linked to a concrete, or psychic equivalent mode of functioning (Fonagy et al., 2016). Hypermentalizing has, instead, been described as the opposite tendency, that of developing very complex models of the mind that, nevertheless, have little or no correspondence to observable evidence (Fonagy et al., 2016). The latter relate to a pretend mode of functioning, characterised by elaborated discussions about mental states with no understanding of what it is like to have and/or feel these mental states.

A lack of parental reflective function has been associated with disrupted attachment in childhood, impaired mentalizing and later life psychopathology (Fonagy et al., 2002; Fonagy et al., 1998), including difficulties recognising, naming and describing emotions (alexithymia), difficulties with symbolic capacity, and difficulties in affect regulation and impulsive behaviours in the offspring (Fonagy et al., 1998). Developmentally, it has also been associated with inflexible pre-mentalistic modes of functioning in the growing child and in the mature adult. These pre-mentalistic modes of functioning (extensively reviewed in Cucchi, 2016) are characterised by a lack of reference to internal states when reflecting upon behaviour and a concrete, inflexible thinking style which together translate into the concretisation of inner life. In this regard, Skårderud (2009) p.72 warns that: ‘When mentalizing is impaired, the body may take on an excessively central role for the continuity of the sense of “self”’, particularly relevant to eating disorders and the present research.

Since different levels of mentalizing capacity may influence engagement in psychotherapy (Katznelson, 2014; Bateman & Fonagy, 2006), it is important to clarify whether differences in mentalizing capacity may be responsible for a more treatment-resistant symptomatology. Bateman & Fonagy (2004) argue that traditional psychotherapy takes for granted the individual’s ability for symbolic representation of mental states, presupposing that individuals can appreciate and differentiate their own subjective states from those of the therapist. Such tasks are challenging for individuals who exist in a pre-mentalistic mode of functioning and this difficulty has even led some to suggest that people with impaired mentalizing capacity do not benefit from traditional psychotherapy (Bateman & Fonagy, 2006). It is therefore important to clarify whether individuals presenting with concurrent ED and SH experience impaired mentalization, as difficulties associated with pre-mentalistic modes of functioning can have significant implications for the process of therapy (Bateman & Fonagy, 2004). Skårderud’s claim (2007) that psychotherapeutic approaches with individuals with poor mentalizing capacity should specifically focus on the rehabilitation of this function would therefore be substantiated.

While hitherto the mentalization literature has concentrated on Borderline Personality Disorder- BPD (Fonagy, 1999; Fonagy & Bateman, 2008a; Fonagy & Bateman, 2008b; Fonagy & Luyten, 2009), Skårderud (2007) has suggested the concrete thinking style and lack of reference to internal states when explaining behaviour present in ED reveals a lack of a mentalizing stance in that group also. Indeed, individuals presenting with Eating Disorders also display significantly lower Reflective Functioning (RF) than other Axis I disorders (Fonagy, 1999; Ward et al., 2001; Müller et al., 2006; Rothschild-Yakar et al., 2010).

Interestingly, the literature has also suggested the existence of pre-mentalistic states of mind in individuals who engage in self-harm (SH); (Farber et al., 2007; Lane, 2002; Bateman & Fonagy, 2012) and researchers have highlighted shared demographic and phenomenological characteristics between ED and SH pathology (Favaro & Santonastaso, 2000). The last point has prompted Favaro & Santonastaso (2000) to argue that the relationship between the two pathologies goes beyond a pure statistical correlation. Comorbid SH in individuals with ED is a poor prognostic factor (Favaro & Santonastaso, 2002) and correlates with more severe and treatment-resistant eating disorder symptomatology (Claes, Vandereycken & Vertommen, 2003; Peterson & Fischer, 2012; Fujimori et al., 2011; Muehlenkamp et al., 2011; Islam et al., 2015; Olatunji et al., 2015). Anorexia Nervosa (AN) and Bulimia Nervosa (BN) have been the main focus of attention for clinicians and researchers (Rikani et al., 2013). However, the term “eating disorders” covers a multitude of presentations that range from food-intake restriction, to emaciation, frequent binge eating, and vomiting, occurring either together or separately. Whilst these conditions can have different features and presentations, people affected by them share a severe disturbance in eating patterns and related thoughts and emotions, and tend to become pre-occupied with food and body weight (American Psychiatric Association, 2013).

A recent meta-analysis has confirmed significant comordibity between ED and SH (Cucchi et al., 2016). Whereas  Lacey & Evans (1986) had proposed the existence of a “multi-impulsive personality disorder” and argued that impulsivity mediates the relationship between ED and SH, others suggested a “repetitive self-mutilation syndrome” in which self-harm acts alternate with other behaviours such as ED ( Favazza & Rosenthal, 1993). However, by grouping SH with other impulsive behaviours such as substance misuse, these authors fail to explain the specific link between ED and SH and ignore contradictory findings suggesting that substance misuse significantly predicts lower rather than higher SH rates (Cucchi et al., 2016). Furthermore, factor analyses suggest the existence of “compulsive”, as well as of “impulsive” aspects to SH (Favaro & Santonastaso, 1998; Favaro & Santonastaso, 2000; Favaro & Santonastaso, 2002), indicating that there might be more to the ED/SH relationship than impulsivity. Given that the literature suggests that together with the capacity to conceive of mental states in symbolic terms and to mentalize comes the ability to modulate affect (Skårderud, 2009), Cucchi (2016) hypothesised that the absence of reliable internal self-regulation might cause some vulnerable individuals to feel out of control internally. This would result in an alienation between the psyche and soma which would leave the body to carry the burden of emotional expression (Lane, 2002). A stronger need to control the body, displayed by resorting to both ED and SH may be associated with lower mentalizing abilities, in particular hypomentalizing. In fact, the available literature suggests a concreteness of symptoms and thinking styles in both individuals who engage in ED and SH that seems to preclude hypermentalizing. Furthermore, both ED and SH have been linked to alexithymia (Nowakowski, McFarlane & Cassin, 2013; Norman & Borrill, 2015) and a link between hypomentalizing and SH was reported in a community sample (Badoud et al., 2015). Moreover, a link between alexithymia and hypomentalizing has been reported in other functional somatic disorders (FSS) (Luyten et al., 2012). Hence, we hypothesise that the clinical groups will score low on certainty about mental states and high on uncertainty about mental states. In addition, considering the above, we hypothesise that the comorbid group will display more hypomentalizing that the non-comorbid group. If true, it could partly explain the more severe presentation that individuals who present with concurrent ED and SH exhibit.

Given that the journey towards a conceptualisation and an understanding of what reflective functioning is has only recently started, it is helpful to investigate how the RFQ relates to associated concepts. In the present study we aimed to explore the relationship between reflective functioning and four other similar concepts. As well as alexithymia, other overlapping concepts form a tangled web encompassing the territory of mentalization. These concepts have been described as “conceptual cousins”, since none share the exact same boundaries (Allen, Fonagy & Bateman, 2008). For example, although it has been argued that the cognitive element of mentalizing is also addressed by the theory of mind (ToM) (Baron-Cohen et al., 2001) and that empathy taps into the emotional aspect of mentalizing (Allen, Fonagy & Bateman, 2008), it can be argued that their domain is narrower. In fact, whilst the literature on theory of mind addresses the cognitive development of mentalizing, arguably it fails to encapsulate the relational and affect regulative aspects of interpreting behaviour (Allen & Fonagy, 2007) and can be thought of as “cold knowledge” (Gorska & Marzal, 2014); it also focuses primarily on others, rather than on the self. Similarly, the domain of empathy is slimmer compared to the concept of mentalizing, as empathy often entails empathy for others, rather than for the self. Instead, it has been argued that the “curious” attention for both the cognitive and emotional aspect of psychological states that mentalizing endorses can be encapsulated by the concept of “mindfulness” (Allen, Fonagy & Bateman, 2008) although, again, mindfulness can be differentiated for its exclusive focus on the present. Instead, mentalizing adopts this stance also towards the past and the future. Hence, exploring those notions and how they relate to mentalizing can help clarify the complex concept that the current study aims to investigate by delineating its various facets and its relationships to related terms. Exploring these notions can also contribute to a better understanding of the theoretical framework surrounding mentalizing.

The present study therefore proposes to utilise the new RFQ to address the following questions:

(a) whether individuals with ED display lower mentalizing abilities than a control group, hence confirming Cucchi’s (2016) results

(b) whether individuals who present with ED show greater difficulties arising from hypo-, rather than hyper-mentalizing relative to control

(c) whether individuals with comorbid ED and SH differ in mentalizing skills from individuals with ED only.

(d) whether individuals with comorbid ED and SH report more hypo-mentalizing than their non-comorbid ED counterpart.

(e) what is the relationship across these groups between mentalizing and the related concepts of alexithymia, theory of mind, empathy, and mindfulness.

**Figure 1:**
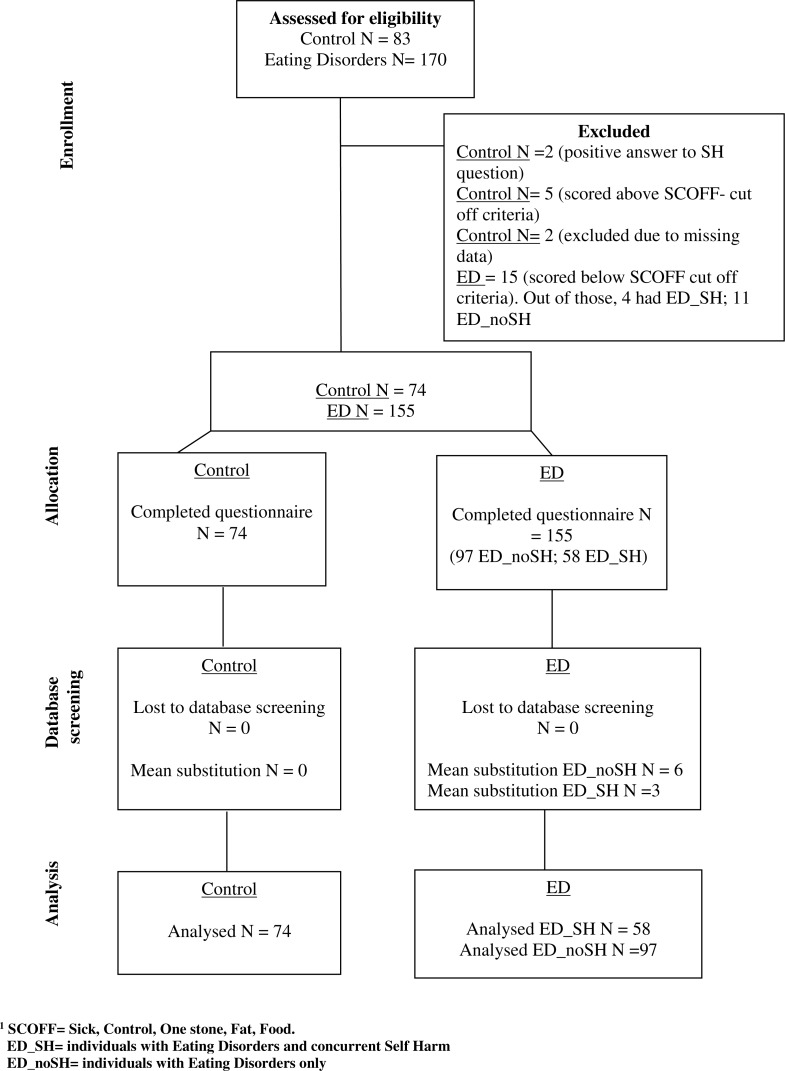
Consort diagram.

## Method

### Design

The design was quasi-experimental, cross-sectional and questionnaire-based. Mentalization was compared in individuals presenting with ED and SH (ED_SH), ED without SH (ED_noSH) and a healthy control group. The quasi-experimental independent variable (IV) was group-type (ED_SH; ED_noSH; Control) and the dependent variables (DV) were reflective function (RFQc and RFQu), and measures of alexithymia, theory of mind, empathy, and mindfulness. The primary interest was in reflective function, with the remaining DVs providing validation and context to this new variable.

### Participants

The ED group initially consisted of a total of 186 individuals (Fig. 1). Of those 186 who started the online survey from different worldwide countries, 115 completed it. An additional 55 people were recruited through an eating disorders unit in the UK National Health Service after being diagnosed by a clinician with either AN or BN according to DSM 5. A history of self-harm was assessed via self-report. This brought the number of the ED group with and without concurrent SH to 170. Inclusion criteria assessed via self-report were: ED symptomatology, age between 18–65, and English-speaker. Exclusion criteria assessed via self-report were: current suicidality, inpatient status, or presence of cognitive impairments/psychotic illnesses. The non-clinical control group was recruited through word of mouth, online advertisement and social networks; 116 people in the control group started the questionnaire online of whom 83 people completed it. Exclusion criteria for this group were the presence of current psychological difficulties and those under 18 years of age. In addition, the non-clinical group was screened for possible ED pathology based on the SCOFF questionnaire (Sick Control One Stone Fat Food—SCOFF, Morgan, Reid & Lacey, 1999) and for positive answers on the SH question. After further exclusion of participants owing to missing data, the numbers in each group were Control *N* = 74 (48 female), ED with SH *N* = 58 (40 female) and ED without SH *N* = 97 (80 females). Our ED sample had rather more SH (37%) than might be expected from reported prevalence (27%) (Cucchi et al., 2016). While unexplained, this distribution enabled a more powerful test of the hypotheses to be made.

Recruitment took place at a specialist NHS Eating Disorder Service, through UK and USA associations offering support and information to people with ED and with the collaboration of 2 professionals who worked privately.

### Ethical considerations

The current research only included participants over 18 years of age. Potential participants were informed of the nature and goals of this research via a Participant Information Sheet presented either as a separate paper copy (as was the case for the NHS participants), or as an introduction incorporated in the online survey (remaining participants). Participants were given the contact details of the researcher if they wished to ask further questions. Those happy to proceed gave their written consent either on paper or electronically. Participants were reminded of their right to withdraw at any stage of the process. Anonymised data was kept on a password-protected electronic database. Ethical approval was obtained from the National Research Ethics Service (NRES) -ID number 10/H1102/60, Beat eating disorders, Somerset and Wessex Eating Disorders Association and City University London’s Ethics Committee.

### Demographic details

Self-reported details were collected for each participant on gender, age, relationship status, ethnicity, employment status, education and occupation (Table 1).

**Table 1:** Demographics for the three groups, with tests for group differences with effect size and *p* values.

**Category**	**Sub category**	**ED_SH**		**ED_noSH**		**Control**		**Test of group difference**	**Effect size/*p* value**
		**Mean**	**SD**	**Mean**	**SD**	**Mean**	**SD**		
Age	(in years)	28.4	10.6	33.3	12.4	37.8	9.7	*F*(2, 226) = 11.4	${\eta }_{p}^{2}=.09$, *p* < .001
		**N**	**%**	**N**	**%**	**N**	**%**		
Gender	Female	40	69	80	82.5	48	64.9	*χ*^2^(2) = 7.4	*p* = .02
	Male	18	31	17	17.5	26	35.1		
Relationship status	In a long-term relationship	20	34.5	34	35.1	47	63.5	*χ*^2^(2) = 16.7	*p* < .001
	Not in a long-term relationship	38	65.5	63	64.9	27	36.5		
Ethnicity	White British/Irish	45	77.6	70	72.2	24	32.4	*χ*^2^(4) = 42.3	*p* < .001
	Any other white background	8	13.8	21	21.6	26	35.1		
	Non-white background	5	8.6	6	6.2	24	32.4		
Employment	Employed	22	37.9	46	47.4	45	60.8	(Unemployed vs. other)	
	Self-employed	3	5.2	12	12.4	7	9.7	*χ*^2^(2) = 12.7	*p* = .002
	Unemployed	16	27.6	11	11.3	5	6.9		
	Studying	16	27.6	25	25.8	14	19.4		
	Retired	0	0	1	1	3	4.2		
	Homemaker	0	0	0	0	0	0		
Education	Secondary school to age 16	4	6.9	2	2.1	4	5.6	Indep. samples median test	*p* = .002
	Secondary school to age 18	19	32.8	24	24.7	11	15.3		
	Non-degree vocational work- based training	9	15.5	15.5	15.5	7	9.5		
	University degree	15	29.9	36	37.1	21	27.8		
	University postgraduate degree	8	13.8	16	16.5	20	28.4		
	University doctoral level	2	3.4	4	4.1	11	15.3		

**Notes.**

ED_SH Eating Disorder with Self Harm ED_noSH Eating Disorder without Self Harm

### Questionnaires

#### Mentalization

Mentalization was measured using the Reflective Functioning Questionnaire (Fonagy et al., 2016; Badoud et al., 2015) which comprises eight items and includes two subscales: certainty (RFQc) and uncertainty (RFQu) about mental states. Fonagy et al.’s (2016) original validation paper describes how the original 6 point Likert scale was changed to a 7 point scale in order to increase the range of scores. The present study adopted the recommended final 7 point Likert scale which contains answers ranging from strongly disagree to strongly agree. Non-linear recoding of the scale responses is used to measure response strength on just one of the ends of the scale (see Table 2). Of the 6 items on each subscale, two are unique and four shared across the two scales. With the RFQc subscale, the extent to which an individual is certain about mental states is measured by how much they disagree with statements such as ‘People’s thoughts are a mystery to me’. The items are rescored (3, 2, 1, 0, 0, 0, 0 with 3 = disagree strongly) such that strong disagreement reflects hypermentalizing, and agreement to any degree (or a neutral response) reflects more genuine mentalizing (acknowledging the opaqueness of mental states). With the RFQu subscale, uncertainty about mental states is measured by how much the individual agrees with statements such as ‘Sometimes I do things without really knowing why’ and is rescored (0, 0, 0, 0, 1, 2, 3; with 3 = agree strongly). High scores reflect a stance characterised by a lack of knowledge about mental states, or ‘hypo-mentalizing’, and lower scores represent an acknowledgement of the opaqueness of mental states, a characteristic of good mentalizing. Both scales are based on a mean of the 6 items.

**Table 2:** Recoding of the RFQ. The first 4 items are used in both scales, but in opposite directions.

Item		Coding for RFQc (Certainty = Hypermentalising)							Coding for RFQu (Uncertainty = Hypomentalising)						
	RESPONSE:	Strongly Disagree…………Strongly Agree							Strongly Disagree…………Strongly Agree						
		1	2	3	4	5	6	7	1	2	3	4	5	6	7
RFQ2	I don’t always know why I do what I do	3	2	1	0	0	0	0	0	0	0	0	1	2	3
RFQ4	When I get angry I say things that I later regret	3	2	1	0	0	0	0	0	0	0	0	1	2	3
RFQ5	If I feel insecure I can behave in ways that put others’ backs up	3	2	1	0	0	0	0	0	0	0	0	1	2	3
RFQ6	Sometimes I do things without really knowing why	3	2	1	0	0	0	0	0	0	0	0	1	2	3
RFQ1	People’s thoughts are a mystery to me	3	2	1	0	0	0	0	NOT USED						
RFQ3	When I get angry I say things without really knowing why I am	3	2	1	0	0	0	0	NOT USED						
RFQ7	I always know what I feel.	NOT USED							3	2	1	0	0	0	0
RFQ8	Strong feelings often cloud my thinking	NOT USED							0	0	0	0	1	2	3

Because of the four shared items, scored in opposite directions, while each scale runs from 0 to 3, the sum of the two scales cannot exceed 4. There is consequently a strong negative correlation between the two subscales, consistent with their opposing interpretations of hypo- and hyper- mentalizing.

#### Eating disorders

The SCOFF (Sick Control One Stone Fat Food—SCOFF; Morgan, Reid & Lacey, 1999) questionnaire is a psychometrically valid 5-item screening test (Botella et al., 2013) which indicates the possible presence of an eating disorder when more than 2 yes responses are given. The measure was designed to suggest a likely case rather than a diagnosis and as such it arguably lacks clinical specificity. However, its statistical validity is sufficient for it to be used routinely in all individuals considered at risk (Morgan, Reid & Lacey, 1999) and based on the reported false positive figures of 12.5% (Morgan, Reid & Lacey, 1999), 14 out of the ED internet sample might have been wrongly included. This equates to only 8% of the total sample size, which seemed an acceptable trade off.

#### Self-Harm

A single question was added to page 5 of the SCOFF questionnaire: “During the past week have you deliberately hurt yourself without meaning to kill yourself (e.g., cut yourself, burned yourself), punched yourself, put your hand through windows, punched walls, banged your head?)”, with a simple Yes/No answer.

#### Alexithymia

Toronto Alexithymia Scale TAS-20 (Bagby, Parker & Taylor, 1994) measures alexithymia in 3 factors including difficulty identifying feelings and distinguishing them from bodily sensations, difficulty describing feelings, and externally-oriented thinking. The scale comprises 20 items, five of which are reversed, which are scored using a 5-point Likert scale. The total alexithymia score is the sum of all 20 items. The TAS-20 uses cut-off scoring: equal to or less than 51 = non-alexithymia, equal to or greater than 61 = alexithymia. Scores of 52 to 60 = possible alexithymia.

#### Theory of mind

The “Reading the Mind in the Eyes” Test, RMET (Baron-Cohen et al., 2001), while originally devised to assess theory of mind in people with autism, has recently been used to successfully differentiate mentalizing skills in BPD (Frick et al., 2012). Participants are presented with 36 photographs of the eye region of faces and are asked to interpret the person’s emotional state by choosing from four words describing feelings. The test is scored by summing all the correct answers. Scores below 22 indicate difficulty inferring mental states. In the original administration of the RMET by Baron-Cohen et al. (2001) the stimuli were presented with a set of eyes on each page and a glossary to clarify less commonly used words (i.e., aghast, despondent). Instead, in the current study, the RMET was the last of the five questionnaires and was presented with six set of eyes on each page. No glossary was provided.

#### Empathy

The Interpersonal Reactivity Index, 7-item Perspective Taking Subscale (Davis, 1983), was used to measure cognitive empathy, the tendency to spontaneously adopt the psychological viewpoint of others. The PTS uses a 5-point Likert scale and has good construct, predictive criterion and divergent construct validity (Davis, 1983)

#### Mindfulness

The Kentucky Inventory of Mindfulness Skills, KIMS (Baer, Smith & Allen, 2004), ‘Describing’ and ‘Acting with Awareness’ subscales were administered. The scales consist of 18 statements (nine reversed score/polarised items and nine normally polarised items) on a 5-point Likert scale with a maximum score of 90.

#### Impact of psychotherapy

To control for any possible influence of psychotherapy, participants were asked to state whether they had any previous experience of psychological therapy and if so, for how long.

### Research hypotheses

In the course of this study, two analyses were run: one testing for group differences and the other looking to cross-validate the RFQ against other measures.

#### Comparing three groups on mentalizing ability on the RFQc

We hypothesized that poor mentalizing will characterize both ED groups relative to Control. In addition, those with comorbid ED and SH were predicted to have additional difficulties with mentalizing beyond those in the ED with no SH group. In terms of the RFQc and RFQu subscales, weak mentalizing ability was operationalised here as hypo-mentalizing, indicated by high scores on RFQu (Uncertainty).

#### Exploring the relationship between mentalizing ability and other scales and measures of related constructs within the whole sample

We hypothesized that the degree to which an individual is hypo-mentalizing will show a positive correlation with alexithymia and negative correlations with measures of mindfulness, empathy and theory of mind.

### Statistical analysis

The data were analysed using SPSS (SPSS IBM, Armonk, NY, USA). Data were screened for anomalous entries, consistency with inclusion/exclusion criteria and missing values.

Group demographic differences were explored using Chi-square tests for categorical variables (e.g., gender), and Kruskal–Wallis and Wilcoxon tests for ordinal data or data non-normally distributed within groups (i.e., age and educational attainment).

Differences in mentalizing ability between the three groups were investigated using a MANOVA with RFQc and RFQu as the dependent variables, and the three groups as the between subjects independent variable. In addition, the statistically significant results of the MANOVA were followed up with a Linear Discriminant Analysis, to look specifically at the practical significance of the results for differentiating SH from non-SH within the ED group.

The relationship between hypo-mentalizing and related concepts was explored using Pearson’s correlation r and confirmed with non-parametric tests.

## Results

### Part 1: group comparison using the RFQ

#### Reliabilities analysis

All validated scales that were used were checked for reliability, and satisfactory levels of Cronbach’s alpha were found (.7 or greater).

#### Comparing 3 groups on mentalizing ability using the RFQ

**Figure 2:**
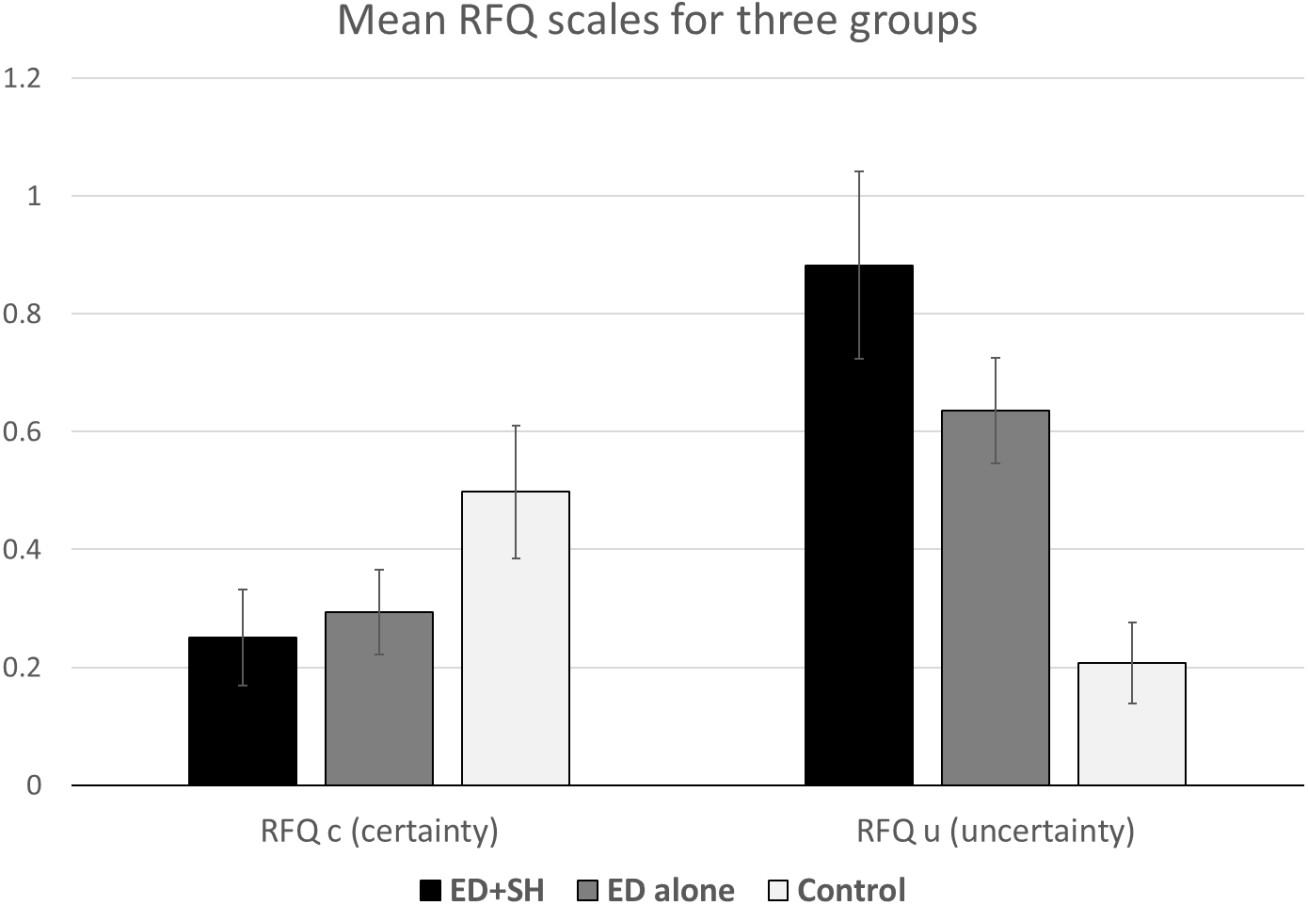
RFQc and RFQu mean scores across the three groups. Error bars show 95% CI.

The RFQu measures hypo-mentalizing and high values on this scale indicate high uncertainty about mental states, hence difficulties with mentalizing. The scale ranges from 0 to 3. For the RFQu, individuals with ED_SH scored highest (*M* = 1.59, *SD* = .82), followed by the ED_no SH group (*M* = 1.27, *SD* = .65), who, in turn, scored higher than the control group (*M* = 0.52, *SD* = .54) (see Fig. 2). The RFQc measures hyper-mentalizing, where extreme high scores on this scale (which also ranges from 0 to 3) can indicate an unrealistic degree of certainty in respect to mental states. On the RFQc individuals presenting with ED_SH (*M* = 0.54, *SD* = .53) scored lower than individuals with ED no SH (*M* = 0.65, *SD* = .58), who in turn scored lower than controls (*M* = 1.03, *SD* = .73). The mean for the controls suggests that a certain low level of disagreement with the statements in the RFQc is consistent with normal mentalizing function. That the ED groups scored lower than controls can be seen as a consequence of their higher scores on RFQu, and the negative correlation between the scales.

A one-way MANOVA with the three groups as Independent Variable and the two subscales as Dependent Variables revealed a significant effect of Group, Pillai’s *V* = .296, *F*(4, 452) = 19.6, *p* < .001, ${\eta }_{p}^{2}=.15$. In addition, separate univariate ANOVAs on the two outcome variables revealed two significant effects: RFQc, *F*(2, 226) = 12.06, *p* < .001, ${\eta }_{p}^{2}=0.10$ and RFQu, *F*(2, 226) = 47.2, *p* < .001, ${\eta }_{p}^{2}=.30$.

For the RFQc scale, pairwise comparisons confirmed that the control group differed significantly from both the ED_no SH and from the ED_SH (both *p* < .001). The two ED groups did not differ (*p* = .26). For the RFQu scale all three groups were significantly different (all *p* < .005).

#### Confounding variables

Two regression analyses were run to check for confounding variable effects, one predicting RFQc, and one predicting RFQu. The diagnostic groups were coded as two dummy variables—one representing ED_SH versus the rest, and the other representing ED no SH versus the rest. The following seven demographic and incidental variables were then included as predictors. Ethnicity: to achieve roughly equal cell sizes, ethnicity was recoded into three categories, British/Irish white (139), non-British white (55), and other (35). Dummy variables were created for the first two categories. Gender, relationship status, and whether the client was in therapy were included as binary variables, and age and educational attainment as interval level scales. Employment status was coded as unemployed (1) or other (0). The regression model for RFQc was significant (Adjusted *R*^2^ = .10). Within the predictors, diagnostic group variables were significant (beta = .23 and .19, *p* < .05), and no other variables reached significance. For RFQu, prediction was better (Adjusted *R*^2^ = .31). Diagnostic group was strongly predictive (beta = .53 and .45, *p* < .001), and no other variables were significant, with the exception of Unemployed status (beta = .13, *p* = .03). It can be concluded that none of the above variables were responsible for the differences observed between groups in RFQ values.

#### Comparing the online self-reported and the clinician-diagnosed sample

**Figure 3:**
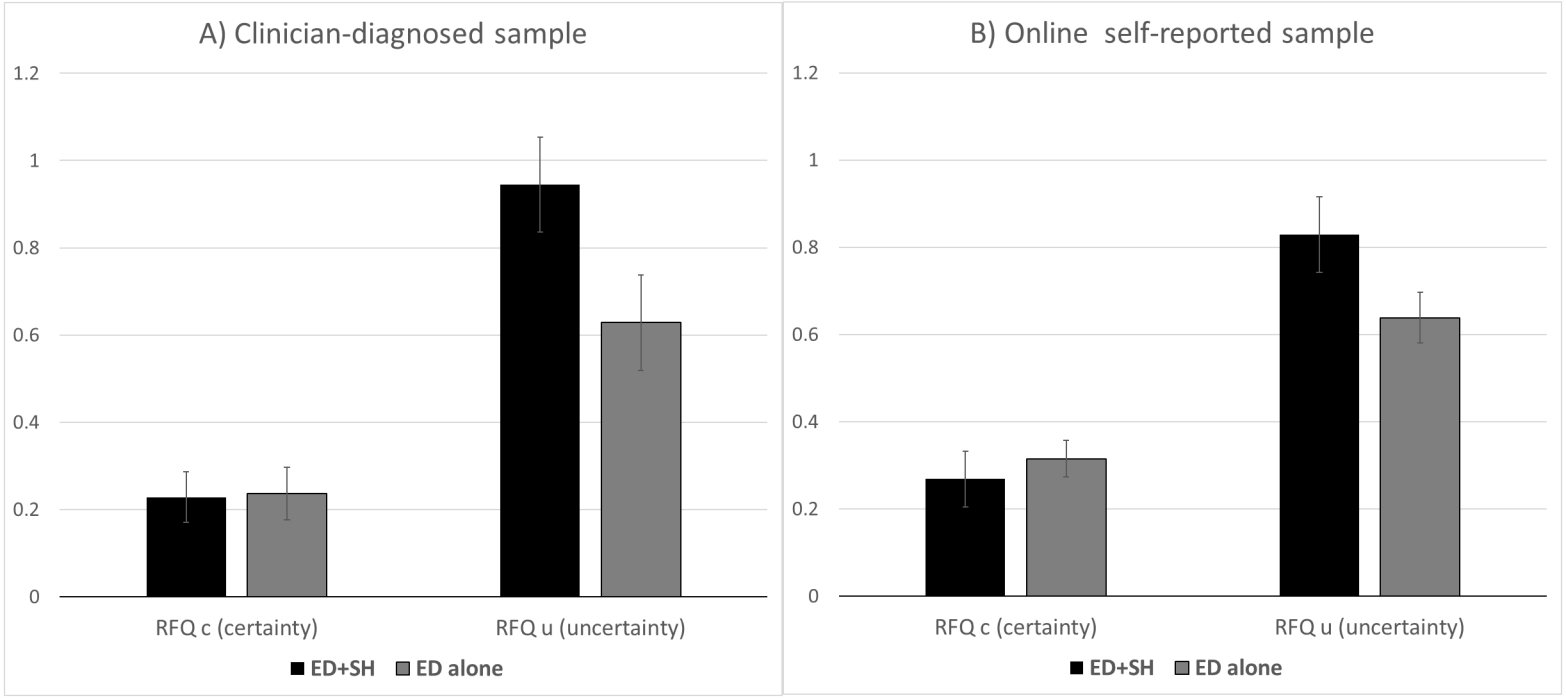
Means for RFQc and RFQu for the online self-reported and the clinician-diagnosed samples. Error bars show Standard Errors. (A) Clinician-diagnosed sample; (B) online self-reported sample.

Given the heterogeneity of the group with self-reported ED symptomatology which was largely recruited through third-sector organisations, we repeated the main analysis comparing the online self-reported group to the ED sample of 53 individuals who were recruited through an eating disorders unit in the UK NHS after being diagnosed by a clinician according to DSM 5. Figure 3 shows the means for the two ED groups. Because of reduced power, the statistical significance of the effect of Group (ED_SH vs ED no SH) within each sample was no longer significant. However it is clear from the figure that the same pattern of data was seen in each sample, with differences in means in the clinician-diagnosed sample being slightly more extreme.

#### Discriminating SH within the ED sample

To investigate the clinical significance of the main result, discriminant analysis was used to predict self-harm in the ED group using the two subscales of the RFQ. The function was statistically significant (*p* = .02), and using cross-validation 89% of non-harmers were correctly predicted, but only 21% of self-harmers. This result implies that the RFQ may have some value as a screening instrument for identifying those NOT at risk of self-harm. Canonical Discriminant Function Coefficients revealed that the RFQu loaded highly on this function (*r* = 1.05), but the RFQc did not (*r* = 0.12).

To see if predictability could be improved with the addition of other information about a person, a stepwise discriminant function analysis was performed with variables including age, gender, relationship status and personal therapy. Age was the second best predictor after the RFQu (*λ* = .91, *F*(2, 151) = 7.2, *p* ≤ .001). No other variables were significant. When age was included in the analysis, the predictive value of the scale for identifying self-harmers increased to 31%, or 28% on the cross-validated measure. To be specific, with cross validation, 16 of the 58 self-harmers and 84 of the 97 non self-harmers were correctly identified by the analysis. The implication is that the non self-harming group are more consistent on the RFQu scale, and so are easier to identify. This interpretation is supported by the greater variance in the self-harm group (*SD* = .78 versus .57 for non self-harmers).

#### Exploring convergent validity between the new RFQ and allied concepts (alexithymia, theory of mind, empathy and mindfulness)

Pearson’s correlation was used to explore the relationship between the RFQ and related concepts. Table 3 shows our findings. Because of their negative correlation, RFQc (certainty) and RFQu (uncertainty) correlated with the other variables in opposite directions. Alexithymia (TAS) was significantly positively correlated with uncertainty (RFQu, *ρ*(199) = .66, RFQc, *ρ*(199) =  − .46, both *p* < .001). Theory of Mind (RMET) was not significantly correlated with either scale (*p* > .10). Empathy (PTS) and Mindfulness (KIMS) were both negatively correlated with uncertainty (for PTS with RFQu, *ρ*(209) =  − .37, and with RFQc, *ρ*(209) = .28, and for KIMS, with RFQu, *ρ*(204) =  − .56, and with RFQc (*ρ*(204) = .39, all *p* < .001). Analysis with nonparametric correlations confirmed the pattern of results.

**Table 3:** Correlations (Pearson *r*) between the RFQ and related measures.

	RFQu	PTS	KIMS	TAS	RMET
RFQc	−.608^**^	.288^**^	.397^**^	−.469^**^	−.140^*^
RFQu		−.379^**^	−.561^**^	.668^**^	.044
PTS			.180^*^	−.335^**^	.123
KIMS				−.707^**^	−.042
TAS					.021

**Notes.**

** *p* < .001.

* *p* < .05.

In sum, as would be expected, hypo-mentalizing was associated across the full sample with difficulties in reading emotions (alexithymia), and low scores for empathy and mindfulness. Interestingly there was no evidence for an association with Theory of Mind.

When mentalizing was correlated with length of therapy, no significant results were found, confirming current empirical evidence (Levy et al., 2006; Karlsson & Kermott, 2006; Vermote et al., 2010).

## Discussion

The current study aimed at clarifying findings from previous research that sought to investigate whether there are significant differences in mentalizing abilities amongst groups with ED with or without concurrent SH, compared to controls. The strength of the present research is that it uses the newly validated version of the RFQ (Fonagy et al., 2016), hence adding to previous research (Cucchi, 2016) with a more robust measure.

The RFQc and the RFQu are intended to tap into opposite dimensions of mentalizing: hyper-mentalizing and hypo-mentalizing respectively. This explains how the 3 groups scored on opposite trajectories on the two subscales. Results showed that individuals with ED_SH are most prone to hypo-mentalizing. It can be argued that this conclusion is in line with current literature that describes concrete thinking styles in narratives of individuals who present with ED and SH alike (Skårderud, 2007). Indeed, hypo-mentalizing might explain this client group’s lack of knowledge about mental states and extreme difficulty in describing others in terms of mental states. This is also confirmed by the RFQu’s positive correlation to the TAS, a measure of alexithymia, implying a link between hypo-mentalizing and alexithymia.

In terms of the relationship between the subscales of the RFQ and its allied constructs, results suggest the RFQu shows a positive correlation with alexithymia and a significant negative relationship with the measures of empathy and mindfulness. It could be hypothesised that the RFQu, whose higher scores measure hypomentalizing, taps into individuals’ failure to understand mental states, which goes hand in hand with failure to understand emotions, lack of empathy and lack of mindfulness. The negative correlation between mentalizing and alexithymia suggests that having a solid emotional vocabulary is essential to the development of a reflective function. In contrast, contrary to our hypothesis, mentalizing as evaluated by the RFQ did not produce a significant correlation with the theory of mind. This could arguably be because the RFQ and the RMET tap into different aspects of the same construct; the former tapping into a more emotional aspect, and the latter tapping into a more physical aspect (reading physical features).

The implications of this study’s findings corroborate previous research (Badoud et al., 2015; Cucchi, 2016) which confirms an association between hypomentalizing and self-harm. Whereas this association was previously reported in a community-based study (Badoud et al., 2015), the present study is highly suggestive that a similar finding would be found in a clinical group also. As the present study featured a mixture of participants with self-reported and clinician-diagnosed ED symptoms, our conclusions must be qualified. Nonetheless, comparing the 53 NHS patients who had a clinician-rated ED diagnosis with the online self-reported group revealed a similar pattern albeit non-significant most likely due to the underpowered analysis. The current research is highly suggestive that a history of eating disorder and concurrent self-harm is associated with the lowest levels of certainty and highest degrees of uncertainty about mental states, which might explain the lack of reference to internal states present in narratives of individuals who engage in ED and SH behaviours (Bateman & Fonagy, 2004; Bateman & Fonagy, 2006; Skårderud, 2007). The literature reminds us that an important indicator of good mentalizing is an attitude of curiosity towards mental states, coupled with an awareness of their opaqueness (Allen & Fonagy, 2007). The literature further suggests that a certain degree of certainty on internal consciousness is adaptive (Allen & Fonagy, 2007; Bateman & Fonagy, 2006; Badoud et al., 2015). The present study suggests that, where this functional degree of certainty is absent, individuals might be more susceptible to self-harm (Badoud et al., 2015) and eating disordered behaviours. In addition, the current findings suggest that the presence of both behaviours is linked to the deepest hypo-mentalizing. Interestingly, whereas Badoud et al. (2015) reported a significant correlation between SH and both the RFQc and RFQu on a community-based sample, the present findings suggest that the RFQu alone is able to differentiate mentalization between the ED_no SH and the ED_SH group. Discriminant analysis revealed that the subscale has a good predictive power for non self-harmers, as 89% of non-harmers were correctly predicted. The RFQc was not as effective at predicting SH group membership, even when other variables were included in the analysis. These findings suggest that: (a) the RFQu is more discriminating than the RFQc in determining SH within the ED group, and (b) that the RFQu can quite reliably be used as a screening device to identify those who are not at risk of SH. In fact, where an individual is indicated to be at risk by this screening, there is still only a 11% chance that they will engage in SH.

The RFQu displayed a positive correlation with alexithymia and a negative correlation with empathy and mindfulness, whereas the RFQc displayed a negative relationship with alexithymia and a positive relationship with empathy and mindfulness. These findings shed more light on our initial hypotheses and on the concept of mentalizing as conceptualised by the RFQ and confirm previous research (Badoud et al., 2015). They suggest that mentalizing as measured by the RFQc (or at least where scores fall short of extreme values that would indicate hyper-mentalizing) is a concept more akin to empathy and mindfulness and negatively correlated to alexithymia. In fact, it is arguable that in order to create more complex models of the mind associated with adaptive levels of mentalizing, an individual needs first of all to have an internal emotional vocabulary. Having an internal vocabulary and being able to construct a model of the mind in turn will enable them to recognise and be empathic towards others’ internal experiences as well as being mindful of their own. Instead, hypomentalizing as measured by the RFQu mirrors a lack of attunement and/or knowledge of mental states that is linked to the inability to name, recognise and articulate mental states (alexithymia). Understandably, this way of being makes it difficult for individuals to be mindful of their own internal experiences and feel empathy towards others. Interestingly, none of the subscales showed a correlation with the Theory of Mind measure. This finding, which nevertheless confirms previous results (Taylor, Target & Charman, 2008), might suggest that the RMET and the RFQ rely on different cues for understanding emotions. In fact, whereas the RMET relies on external cues (facial expressions), the RFQ taps into internal states. At the same time, any impact of the way the RMET has been adapted in the current study cannot be ruled out.

### Theoretical implications of the current findings

This study appears to support theorists such as Skårderud (2007) who have suggested that impaired mentalizing might be a core issue in EDs. In the present study both clinical groups presented with significantly lower levels of mentalizing ability compared to non-clinical controls, supporting Skårderud’s claim that in eating psychopathology the body takes on an excessively central role for the continuity of the sense of “self” when mentalizing is impaired. In addition, findings showed that the group with concurrent ED and SH presented with more hypomentalizing difficulties compared to the ED_noSH group. The latter point also seems to support the notion that clients with a stronger need to control the body, displayed by resorting to both ED and SH behaviours, display lower mentalizing capacity. It appears that the deeper the inability to make sense of one’s own and others’ inner experiences and to interpret behaviour according to mental states, the more the focus on the body. More specifically, it seems that the more an individual presents with difficulties with symbolic representations and abstract thinking, characterised by lower mentalizing abilities, the more they seem to concentrate their energies on their immediate physical world, i.e., the body. This indeed appears to support the theoretical notion of the teleological stance of functioning (Fonagy et al., 2002). In fact, as extensively discussed in Cucchi (2016) when operating in the Teleological mode of functioning, expectations concerning agency and change cannot be conceived unless it is in physical terms, prompting individuals to concentrate their energies on their physical world.

An additional theoretical implication originating from the current study relates to the recognised existence of a link between ED and SH. Although impulsivity had previously been deemed to mediate this relationship ( Favazza & Rosenthal, 1993;  Lacey & Evans, 1986), the present findings suggest that mentalizing might also play a role (Skårderud, 2007). In fact, it can be argued that impulse control, together with the capacity to monitor and regulate one’s own emotions, is the product of the development and the organisation of the “self”, which in turn is the result of the development of the capacity to mentalize (Bateman & Fonagy, 2004; Fonagy et al., 2002).

Lastly, the present study indicates the need to address ED and SH simultaneously when the two presentations occur together and the need for the characteristic pre-mentalistic states of mind to be taken into consideration when devising interventions. The latter point supports Skårderud’s argument (2007) that psychological interventions with individuals with compromised reflective functions should be specifically focused on the rehabilitation of this function. Indeed, Katznelson (2014) concluded that there is evidence to suggest that having an understanding of, and being able to think in terms of mental states and how these affect behaviour, has an impact on people’s ability to engage in psychotherapy, a claim echoed by Bateman & Fonagy (2006). In this respect, Katznelson (2014), who reviewed the role of RF as a predictor and mediator of change, concluded that mentalizing can change only through approaches that specifically focus on the integration of split mind states.

Some limitations of this study must be acknowledged. The current research was presented as part of the first author’s Doctoral thesis. Hence time and resource constraints shaped recruitment strategies and the final sample composition. Due to recruitment difficulties in NHS sites, our sampling strategy was widened to include third sector organisations, with a concurrent increase in the heterogeneity of the sample and reliance on patient self-reported diagnostic status. Furthermore, recruitment was carried out mainly through the internet where ED symptomatology was assessed through the use of a screening measure, rather than through a diagnostic interview. Furthermore, assessing self-harm via a single item presented limitations to our analysis. Hence, the main limitation of this study concerns the ED sample used. Whilst it is arguable that individuals who access support from ED organisations experience eating-related symptomatology, the study would have benefitted considerably from the use of a questionnaire such as the EDEQ or EDI-3 in order to assess ED psychopathology in a more rigorous way. Similarly, it would have greatly benefitted from a more detailed assessment of SH behaviour. The relatively heterogeneous clinical sample which included a mixture of online respondents with self-reported diagnoses, together with NHS patients with established diagnoses, raises some questions. It can be argued that this approach could be a source of measurement error and hence pose a problem for data integrity (De Leeuw, 2005). Whilst this is a legitimate argument, the literature also confirms that in recent years sequential mixed-method recruitment strategies, including internet recruitment, have been employed as an effective way to improve response rates ( Dillman et al., 2009). Mixed recruitment methods now constitute an important form of data collection that potentially allow some significant insights to be derived in a resource-limited environment (De Leeuw, 2005). Given the limitations presented by a Doctoral thesis, the mixed-recruitment approach provided a realistic compromise which enabled the research to move forward and promoted the discovery of new findings in a an under-researched topic. Lastly, being self-selected the sample may have been biased by those with less severe psychopathology whose natural interest in the subject had led them to apply and which in turn may have indicated greater psychological-mindedness. Additionally, a preponderance of online recruitment may have precluded individuals less confident with new technologies.

Some limitations of the primary assessment instrument, the RFQ, must be acknowledged. The RFQ measures both a person’s ability to mentalize, but also an individual’s insight into their ability to mentalize. One can speculate whether insight might vary across different conditions and severity of psychological difficulties. Indeed, the literature has often reported that emaciation and the severity of Anorexia Nervosa (AN), or indeed personality disturbances, can be associated with poor psychological insight (Bateman & Fonagy, 2004; Greenfeld et al., 1990). Whether this might have affected individuals’ responses and subsequent performance on the test is worth bearing in mind when conducting future research. Moreover, the issue of psychological insight is extremely pertinent when talking about mentalizing. In fact, whereas some vulnerable individuals might ordinarily display adequate levels of mentalizing, this capacity appears to collapse in the face of stress, especially attachment stress (Allen, Fonagy & Bateman, 2008; Bateman & Fonagy, 2004). It is worth considering that in this study stimuli were administered outside any controlled therapeutic intervention related to the research. Hence one can wonder whether in different circumstances, especially those that stimulate and intensify attachment emotions and the attachment-system, participants might have answered differently. It can also be questioned whether mentalizing abilities might actually change on the basis of whether the emotion mentalized is positive, negative, or neutral. In the present research we have limited our conclusions to hypo-mentalization and its clear association with psychopathologies such as self-harm and eating disorders.

Nonetheless, the current research has a number of significant strengths. First, the study is timely and comes when research on mentalizing in different populations has only recently started. Furthermore, the RFQ is a new measure and little data exists to corroborate Fonagy et al.’s (2016) assertion of its validity as a measure of mentalization. In addition, the fact that participants came from different countries suggests that the relationship ED/SH cuts across geographical boundaries and it is fairly culture-free, implying some “universal” association.

Given that this is the first study of its kind and that it was carried out within the constraints of a Doctoral thesis, the importance of follow up studies appears self-evident. A replication of the current project with more robust and systematic diagnostic measures as discussed above is recommended before definite conclusions can be reached. In addition, particular attention could be given to exploring mentalizing differences in ED subgroups. In fact, given the higher comorbidity rate found between BN and SH, compared to AN, reportedly 33% and 22% respectively (Cucchi et al., 2016), it appears that people who present with BN engage more in SH. Hence further research could explore whether, for example, individuals presenting with BN and concurrent SH display fewer mentalizing skills that those without SH. The same could be investigated in AN groups.

## Conclusions

This study investigated mentalizing differences in individuals presenting with ED with/without concurrent SH compared to non-clinical participants. Despite a number of limitations, our initial hypotheses were supported as individuals who presented with both ED and concurrent SH reported less reflective function than individuals who presented with ED only. In addition, both clinical groups reported significantly less mentalizing ability than controls. Lastly, the present results underscore the importance of routinely screening for comorbid SH in individuals who present with ED and to reflect on the clinical implications that a lack of a mentalizing stance can have on individuals’ ability to engage with the therapeutic process and to initiate change.

##  Supplemental Information

10.7717/peerj.5756/supp-1 Supplemental Information 1 Reflective Functioning Questionnaire, related measures and demographic details

10.7717/peerj.5756/supp-2 Supplemental Information 2 Value labels for the variables in the file

10.7717/peerj.5756/supp-3 Supplemental Information 3 Data

## Additional Information and Declarations

### Data Availability

The following information was supplied regarding data availability: The raw data are provided in a Supplementary File.
