# Supplementary material for: Using the validated Reflective Functioning Questionnaire to investigate mentalizing in individuals presenting with eating disorders with and without self-harm
Source: PeerJ. 2018 Oct 29;6:e5756. doi: 10.7717/peerj.5756 (PMC6211265; doi:10.7717/peerj.5756)
Supplement: Supplemental Information 2 [file peerj-06-5756-s002.zip › File definition.pdf]

```
DATASET NAME DataSet1 WINDOW=FRONT.
DISPLAY DICTIONARY.
```

File Information

```
[DataSet1] C:\Users\sc318\AppData\Local\Microsoft\Windows\Temporary Interne
t Files\Content.Outlook\15LD69CX\AC_database_groups_229.sav
```

Variable Information

Variable	Position	Label	Measurement Level	Role	Column Width	Alignment
ID_number	1	ID: Participant ID	Nominal	Input	22	Left
SH_question	2	During the past week have you deliberately hurt yourself without meaning to kill yourself?	Nominal	Input	8	Right
Group	3	Three groups in the design	Nominal	Input	10	Right
LRFc_s	4	RFQ certainty scale	Nominal	Input	10	Right
LRFu_s	5	RFQ uncertainty scale	Nominal	Input	10	Right
Gender	6	Gender	Nominal	Input	8	Right
Age	7	age in years	Scale	Input	5	Right
Relationship	8	Is the client in a relationship?	Nominal	Input	8	Right
Ethnicity	9	Ethnicity	Nominal	Input	8	Right
EmpStat	10	Employment status	Nominal	Input	8	Right
EduAtt	11	Educational attainment	Nominal	Input	8	Right
Occupation	12	occupation	Nominal	Input	8	Right
Pers_Therapy	13	Has the client received personal therapy?	Nominal	Input	8	Right
No_Pers_Ther	14	Number of months of personal therapy received	Scale	Input	8	Right

Variable Information

Variable	Print Format	Write Format
ID_number	A17	A17
SH_question	F8	F8
Group	F8	F8
LRFc_s	F8.2	F8.2
LRFu_s	F8.2	F8.2
Gender	F8	F8
Age	F8	F8
Relationship	F8	F8
Ethnicity	F8	F8
EmpStat	F8	F8
EduAtt	F8	F8
Occupation	F8	F8
Pers_Therapy	F8	F8
No_Pers_Ther	F8	F8

Variable Information

Variable	Position	Label	Measurement Level	Role	Column Width	Alignment
SCOFF	15	SCOFF	Nominal	Input	10	Right
PTS	16	Perspective taking subscale/Empathy	Scale	Input	10	Right
KIMS	17	Kentucky Mindfulness Inventory	Scale	Input	10	Right
TAS	18	Toronto Alexithymia Scale	Scale	Input	10	Right
eyes	19	Reading the Mind in the Eye	Nominal	Input	10	Right
EatingDisorder	20	Eating disorder group with/without SH	Nominal	Input	16	Right

Variable Information

Variable	Print Format	Write Format
SCOFF	F8.2	F8.2
PTS	F8.2	F8.2
KIMS	F8.2	F8.2
TAS	F8.2	F8.2
eyes	F8.2	F8.2
EatingDisorder	F8	F8

Variables in the working file

Variable Values

Value		Label
SH_question	1	yes
	2	no
Group	1	Control
	2	ED+SH
	3	ED alone
Gender	1	female
	2	male
Relationship	1	yes
	2	no
	3	not stated
Ethnicity	1	white british
	2	white irish
	3	other white
	4	mixed white and black caribbean
	5	mixed white and black african
	6	mixed white and asian
	7	any other mixed
	8	asian indian
	9	asian pakistani
	10	asian bangladeshi
	11	any other asian
	12	black caribbean
	13	black african
	14	any other black
	15	chinese
	16	any other ethnic group
	17	not stated
EmpStat	1	employed
	2	self employed
	3	unemployed
	4	studying
	5	retired
	6	homemaker
EduAtt	1	Secondary School to age 16
	2	Secondary school/college to age 18

Variable Values

Value		Label
	3	Non-degree level vocational work-based training
	4	University degree
	5	University postgraduate studies (e.g. Masters)
	6	University doctoral level studies (e.g. PhD)
Occupation	1	Modern professional occupations
	2	Clerical and intermediate occupations
	3	Senior managers or adminstrators
	4	Technical and craft
	5	Semi-routine manual and service
	6	Routine manual and service
	7	Middle or junior managers
	8	Traditional professional occupations
	9	not applicable
Pers_Therapy	1	yes
	2	no
	3	not stated
EatingDisorder	1	Control
	2	ED both groups
